# Destruction of the Hard Palate, Originating in a Carious Tooth

**Published:** 1855-01

**Authors:** C. Spence Bate


### ARTICLE XIII.

### *Destruction of the Hard Palate, Originating in a Carious Tooth.* By C. SPENCE BATE.

SOME pages back, I had occasion to allude to a case wherein the palate had been destroyed through an abscess, attributed to the abnormal pressure caused by the development of the third molar tooth.

I now wish to draw attention to a somewhat similar case, which was brought under my notice by a lady who consulted me respecting a small hole in the roof of her mouth. This originated in a similar swelling to the last, on the palate, but succeeding severe pain from a carious tooth. The latest which I have heard of this lady, who resides in a distant county, is, that the disease has assumed a somewhat chronic form, a slight discharge still continuing to pass through the hole into the mouth.

The osseous roof of the mouth was eaten through, but the membranes lining the floor of the nose had not been perforated to any great extent; consequently, no passage existed between the two facial orifices which might effect speech or deglutition.

This is by far the most important, as to the results, which I have seen of the kind originating in a diseased tooth; but I have frequently met with cases which must have terminated in exfoliation of the palatal bones but for a timely rescue.

When the membranes existing upon the fangs of the teeth, and those covering the walls of the alveoli, become affected by the pressure of the decayed tooth, it generally develops an abscess. The pus which it originates endeavors to escape, so as to pass out of the system; this it does sometimes through the pulp cavity of the tooth, sometimes between the tooth and the walls of the alveolus; sometimes it works its way out through the external alveolar wall, developing a small abscess or boil upon the surface of the gum. But, should it do neither of these, as may result, particularly if the palatal fang of the upper molars, which sometimes spread out at a large angle with the others, the disease becomes so deep-seated that it is not able to find its way out, and therefore eats through the internal wall of the alveolus, and burrows beneath the palatal integuments, which, in order to fulfil the duties of the mouth, is more than usually tough, and therefore becomes a source of obstruction to the free exit of the pus. The result is that a tumor, increasing towards the base, is formed; it is generally painless, and, when opened, the bone beneath will almost invariably be found denuded of its periosteum.

Of course, in the treatment of such cases, the first and most important step will be, to remove the irritating tooth,—most generally a decayed one, though I have seen it result from a tooth worn by mastication. But, as far as my experience goes, it arises from a tooth planted firmly in the jaw; I much question if it could result from one that was loose, since then the pent-up matter would find its way between the walls of the alveolus and the tooth.

If it should be found that the extraction of the tooth admits of a free passage for the discharge of the pus, there may possibly be no occasion for laying open the tumor; but, if the discharge is still confined, and the operator is certain that pus is formed, the swelling should be freely opened, but with caution, since there is more than one case on record of tumors of similar appearance, upon being lanced, having shown themselves to be traversed by blood-vessels so important, that serious bleeding has been the result, sometimes to the great risk of the patient's life.

Of course, the more systematic treatment courtesy would hand over to the physician; it would properly consist of the iodide of potassium, together with a course of tonics.

These cases, which, from their position, must be looked upon as important, are far from uncommon; and, although their growth in general may be very slow, yet their history, as sometimes detailed to me, has extended over but a few day's duration. One of these last I have but lately seen; the tumor was as large as a pullet's egg, and originated from the stump of a decayed upper lateral, which was removed after some difficulty; the tumor was opened and pus escaped; the bone was found to be denuded of its periosteum to the extent of the surface of a crown piece. An astringent lotion to the part and aperient medicine were ordered, since which I have not seen the individual, who is engaged in the copper works in this locality; and I am informed by others, that he pursues his avocation, and they believe him to be restored.

But the following is a case of more interest to the dentist, inasmuch as it is one wherein the application of artificial teeth was required.

It is about five years since I was sent for by Mrs. J., a widow lady, about 50 years of age, who wished me to make for her an almost entire piece of artificial teeth. Upon examining the mouth previous to taking the model of her gums, I observed a rather extensive swelling of the surface of the palate, chiefly upon the right side of the mesial line, and in front of the mouth existed the fangs of from six to eight teeth.

She told me that she had been conscious of this swelling for about seven or eight years, dating it from a severe tooth-ache which she had in the right lateral incisor, and which had been broken in an attempt made to extract it by her own medical attendant.

The swelling had given her no cause for uneasiness, as it never pained. Occasionally, it increased in size, but always soon again decreased; and this circumstance she attributed to the pressure arising from artificial teeth she had been in the habit of wearing for some time, and which, she imformed me, were well adapted and otherwise comfortable.

Fearing that pressure, particularly of new work, might excite this tumor, I strongly pointed out the very great desirableness of having all the stumps removed, especially that to which she attributed the origin of the mischief, but without obtaining her consent; therefore the pieee was adjusted under existing circumstances, and which she wore for about four years without any injury to the tumor; and it was not until last summer, when, having caught a severe cold, she had much pain in all the stumps, attended with considerable swelling of the gums, that I could obtain her consent to the removal of all the affected teeth, which was done under the partial influence of chloroform. The gums healed, absorption progressed in a healthy manner, and the tumor disappeared. Somewhere about two months was allowed to elapse before fresh models were taken. A fortnight after this, my patient drew my attention to the increase of the old tumor of the palate; this was before any artificial work was applied to the mouth, and therefore could not have originated from undue pressure on the part; but, in truth, it was so very slight, that I imagined that it could never wholly have disappeared, though she strove to assure me to the contrary. I therefore, when the false piece was completed, felt no disinclination to yield to her desire to have them in the mouth. But the swelling, not at all improbably excited by the plate, continued rapidly to increase, until in a few days it rose to such an extent, that I begged her to throw aside her teeth, and place herself under the skillful and experienced hands of my friend Dr. Bird, who laid the tumor open, and found it, he informed me, gorged with blood, there being no escape of matter. Upon the healing of the incisions, the tumor greatly decreased, but had not disappeared when I saw her last, somewhere about a month previous to writing this. Of course, she has not yet been permitted to wear the artificial work.

The remarkable point in this case is, that it should have remained for a period of about twelve years *in statu quo*, neither advancing nor retrograding until the exciting cause (the decayed teeth) was removed,—that it should first wholly disappear, and shortly return with accelerated virulence.

Diseases like these, although fortunately not of every day occurrence, yet are of sufficient frequency to urge upon all the great advantage of preserving the teeth in a healthy condition, or of having them restored as early as possible when they have been attacked by disease; or where this has progressed so far as to render them useless, they should be treated as extraneous bodies, and be forcibly removed, considering it more wise to endeavor to preclude desease than to wait for its presence in order to save a temporary though acute pain.

